# Bacteriological profile and antibiotic susceptibility pattern of common isolates of neonatal sepsis, Ho Municipality, Ghana-2016

**DOI:** 10.1186/s40748-017-0071-z

**Published:** 2018-01-23

**Authors:** Fortress Yayra Aku, Patricia Akweongo, Kofi Nyarko, Samuel Sackey, Fredrick Wurapa, Edwin Andrew Afari, Donne Kofi Ameme, Ernest Kenu

**Affiliations:** 1 Ghana Field Epidemiology and Laboratory Training Programme and Department of Epidemiology and Disease Control, School of Public Health, University of Ghana, Accra, Ghana; 2 Ghana Health Service, Volta Regional Health Directorate, Ho, Ghana

**Keywords:** Neonatal sepsis, Antibiotics, Newborn

## Abstract

**Background:**

Globally, 4 million neonates die annually, with one-third of such deaths occurring as a result of infections. In 2011, there were 7.2million deaths in children below 5 years globally, and a proportion of 40% of these deaths occurred in neonates. Sepsis was reported to account for one-third of these deaths. Presently, multidrug antibiotic resistance is rapidly increasing in Neonatal Intensive Care Units (NICUs), particularly in developing countries and poses a threat to public health. The change in these organisms has been reported to vary across regions, between health facilities and even within the same facility. Continuous surveillance is required to inform antibiotic choice for neonatal sepsis management. We identified the common causative organisms of neonatal sepsis and their antibiotic susceptibility pattern in the Ho municipality.

**Method:**

A cross sectional study was conducted in the Ho municipality from January to May, 2016. A semi-structured questionnaire was used to collect socio-demographic data from mothers of neonates with clinically suspected of sepsis. Clinical data of both mothers and neonates were extracted from case notes. A 2 ml volume of blood was also taken from neonates and dispensed into a 20 ml mixture of thioglycollate fluid broth and tryptone soy broth for culture and antibiotic susceptibility pattern determined.

**Results:**

Out of the 150 clinically suspected neonatal sepsis cases, 91 (60.7%) were males. The Median gestational week was 38 (IQR: 36–39) and Median birthweight was 3.0 kg (IQR 2.5–3.4). The prevalence of culture positive sepsis was 17.3% of the 150 suspected cases. A total of 26 different pathogens were isolated, of which gram positive organisms had a preponderance of 18 (69%) over gram negative organisms 8 (31%). *Staphylococcus epidermidis* was the most common 14 (53.8%) isolate identified. There was a single isolate (4%) each of *Proteus mirabilis* and *Escherichia coli* identified. All the isolates identified showed 100% resistance to ampicillin.

**Conclusion:**

The prevalence of culture proven sepsis was 17.3% and *Staphylococcus epidermidis* was the most common isolate identified. Pathogens isolated were resistant to the first line drugs for management of neonatal sepsis. Hence, the need for a review of first line drug for empirical treatment in neonatal sepsis.

**Electronic supplementary material:**

The online version of this article (10.1186/s40748-017-0071-z) contains supplementary material, which is available to authorized users.

## Background

Neonatal sepsis (NS) is a significant contributor of mortality and morbidity in the newborn [1]. It is estimated that globally 4million newborns die yearly with one-third of the deaths caused by infections [2].Various conditions contribute to high neonatal mortality especially in Sub-Saharan Africa of which neonatal sepsis accounts for approximately 26% [3]. In 2008, 22, 672 deaths were estimated to have occurred among neonates in Ghana with neonatal sepsis causing 4923 deaths (21.7%) [4, 5].

Neonatal sepsis refers to a clinical syndrome that is marked by signs and symptoms of infection in the first 28 days of life, with or without isolation of a pathogen [6]. A normal fetus is sterile until shortly before birth since placenta and amniotic sac serve as effective protection against infection. However, at birth, the newborn is exposed to the microbial environment. NS can be categorized as early onset sepsis (EOS) and late onset sepsis (LOS). EOS is defined as onset of signs and symptoms of infection within 72 h of life and may be associated with pathogen isolation or not. In the LOS, signs and symptoms present after 72 h of life [7] and categorization of EOS and LOS is to show the varying causes and pathophysiology of common isolates related to the time of onset of the condition. It is also crucial in prevention and treatment due to aetiological variation.

Some maternal and neonatal factors have been identified to predispose a neonate to sepsis. In a study on neonatal sepsis conducted in 2013 in China, maternal age > 35; mother with affixed occupation; mother of urban residence; caesarean section delivery; and parity were found to influence early onset neonatal sepsis in a univariate analysis [8]. In a similar study conducted at King Edward Memorial Hospital in Western Australia, it was also found that neonates who were born at a lower gestational age (26 weeks ± 1.8) and those who had a lower birth weight (848 g ± 240 g) developed sepsis [9].

Recent reviews of causative agents associated with infants with sepsis in the developing world revealed that in EOS, gram negative organisms predominated in the ratio of 2:1 with *Escherichia coli* being the most commonly isolated pathogen [10]. However, the main causative agent of LOS in neonatal intensive care unit has been reported to be coagulase negative staphylococcus (CoNS). In a study done in the Aga Khan University Hospital NICU Nairobi Kenya, on 152 neonates who presented with sepsis, also found that 58 (38.2%) of them had LOS; and coagulase-negative staphylococcus was the most common isolate both in EOS and LOS [11]. A similar study done in Tanzania, found that *Staphylococcus aureus* was the most common organism isolated from blood culture and pus swab followed by *Klebsiella* species and *Escherichia coli* [12]. A study conducted in Ghana revealed gram positives as the predominant isolate and included coagulase negative staphylococcus (CoNS), followed by *Staphylococcus aureus* and *Streptococcus* species [13].

The main method of diagnosing sepsis is the isolation of causative agents from blood cultures [14]. Since there are no pathognomonic signs and symptoms for sepsis in neonates, it is necessary to carry out investigations in a well set up laboratory that has the capacity to do so [15]. In a study done in Tanzania, clinical signs such as difficulty in feeding, lethargy, convulsion, increase in respiratory rate and cyanosis had a strong association with culture proven early onset sepsis, whiles hypothermia, chest in-drawing, umbilical redness and jaundice were related to late onset form of sepsis (14). In managing sepsis, neonates are given empirical therapy according to the World Health Organization (WHO) Integrated Management of Neonatal and Childhood Illness (IMNCI) algorithm for early detection of severe diseases including severe bacterial infection [15].

Currently at the Volta Regional and Ho Municipal Hospitals’ NICUs in the Ho municipality, neonatal sepsis accounts for the highest cause of admission and is managed empirically in line with the WHO recommendation of using ampicillin/cloxacillin and gentamicin as first line antibiotics. There is paucity of published data on neonatal bloodstream infection in Ghana and West Africa [13] and over the period, reports show that neonatal sepsis causative agents differ from region to region and vary from time to time [16]. This change in causative agents coupled with their change in response to commonly used antibiotics and the need for periodic health facility based surveys calls for evidence based data was therefore the basis for our study. This study was done to determine the common isolates of neonatal sepsis and their antibiotic susceptibility pattern to commonly used antibiotics.

## Methods

### Study design

The study was a hospital based cross-sectional study carried out at two public hospitals in the Ho municipality (Ho Municipal and Volta Regional Hospitals). A 2 ml volume of blood was taken into a universal bottle for culture from neonates clinically suspected of sepsis and admitted at the two NICUs between January and May, 2016 for culture; to determine the common isolates causing sepsis and antibiotic susceptibility pattern of the isolates.

### Study population

All neonates and their mothers who delivered by either C-section or spontaneous vaginal delivery and did not receive any antibiotic before the operation were included in the study.

For purposes of this study, neonatal sepsis was defined as neonates presenting with one or more of the following features: presence of fever (≥38 °C) or hypothermia (≤36 °C), convulsions, lethargy, difficulty to feed, difficulty to breathe, hypoglycaemia, vomiting, bulging fontanels, respiratory distress, jaundice and signs of infection on the skin and umbilical pus discharge or hypareamia.

### Inclusion and exclusion criteria

All neonates admitted at the NICUs of the two hospitals, who were clinically diagnosed of sepsis by a clinician during the study period, and whose mothers or caretakers consented to be part of the study were included in the study. All neonates admitted at the NICUs of the two hospitals, who were clinically diagnosed of sepsis by a clinician during the study period but died immediately upon arrival before blood culture sample could be obtained, or neonates who were referred to a tertiary facility immediately after assessment were excluded.

### Sample size calculation

Using a prevalence of 11%, a total sample size of 150 was calculated using the Cochrane formula [17]. Out of a total of 250 neonates who were in the Neonatal Intensive Care Units (NICU) during the time of the study, 150 who met the inclusion criteria and were randomly recruited.

### Data collection

Structured questionnaires were used to collect demographic and clinical details of both neonates and their mothers through interview and review of case notes. Data on the pathogens isolated and their antibiotic susceptibility pattern were also collected after laboratory test was done.

### Laboratory investigations

Two millilitres of venous blood was aseptically obtained from the antecubital fossa of each neonate and dispensed into a sterilized universal bottle containing 20 ml of tryptone soy broth to make a 1:10 dilution. In obtaining blood samples, the site was cleaned with isopropyl alcohol concentration of 70% twice before blood was collected. There was strict adherence to microbiological protocol during culture and isolation which included use of sterile loops. In the event of using loops that the authors thought were not sterile enough, isolates were considered as contaminants. Blood culture samples were then transported to the laboratory within 1 h and incubated at 37 °C for 24 h. Each sample was sub-cultured unto commercially prepared blood chocolate agar and MacConkey agar. The sub-cultured agars were incubated at 37 °C and observed for growth. Samples that did not show growth after 24 h were observed for 7 days before regarded as no growth. Pure colonies of samples that showed growth were taken for gram stain test and biochemical tests using commercially prepared reagents. These tests together with characteristic morphology of pure colonies were used for isolation and identification of pathogens.

Antibiotic susceptibilities of isolated pathogens to the selected antimicrobial agents were determined, using the Kirby Bauer disc diffusion method according to the Clinical and Laboratory Standard Institute [18]. Pure colonies of isolates were emulsified to obtain 0.5 MacFarland standard and inoculated on Muller Hinton agar. Antimicrobial discs were place on the inoculated agar and incubated for 24 h at 37 °C; they were then observed for zones of inhibition, breakpoints identified and determined as susceptible, intermediate or resistant according to the Clinical and Laboratory Standard Institute [18]. All cultures received for neonates after an average of 48 h of onset of sepsis was regarded as delayed.

### Data analysis

Case notes were reviewed again and interviews conducted to crosscheck for any anomalies detected. Data collected was checked for completeness and double entry was done. Data was entered into Microsoft Excel and cleaned by crosschecking for missing data, duplicates and outliers. It was then analysed using STATA software version 13.0 (College Station, Texas 77,845 USA). Continuous variables were presented as mean, standard deviation as well as median with inter-quartile range for neonates and their mothers Pathogens isolated from the laboratory investigation and antibiotic susceptibility of the isolates was presented in frequencies and proportions.

### Ethical consideration

Approval for this study was obtained from the Ghana Health Service Ethical Review Committee. Permission was also sort from the Municipal Health Directorate and the hospital administration of the respective hospitals. An informed consent was administered to mothers of neonates before participation in the study, and each respondent was given a unique identifier such that data gathered could not be traced back to respondents. Data was kept safe under lock and key and was accessible only to the principal investigator.

## Results

### Neonatal characteristics

Out of the 150 neonates that were suspected of sepsis and recruited during the study period, 91 (60.7%) were males (Table 1). Majority, 87 (58%) of the mothers of these neonates were delivered by caesarean section. The median gestational week was 38 weeks (IQR: 36–39) weeks; and the median birthweight was 3.0 kg (IQR 2.5–3.4) kg. Most of the deliveries, 109 (72.7%) were either at the Volta Regional Hospital or Ho Municipal Hospital; (Table 1). Of the 41 born outside the study facilities, 11 (26.8%) were home deliveries.

**Table 1:** Characteristics of neonates with suspected sepsis on admission at NICU, Ho municipality, 2016

Variable	Count (N)	Percent (%)
Sex		
Male	91	60.7
Female	59	39.3
Birth weight		
< 2500 g	35	24.5
≥ 2500 g	108	75.5
Place of birth		
Within study facilities	109	72.7
Outside study facilities	41	27.3
Sepsis category		
Early onset sepsis	81	54
Late onset sepsis	69	46
Gestational age		
≤ 36	41	27.3
37–40	94	62.7
≥ 41	15	10
Length of hospital stay		
≤ 7 days	77	51.3
≥ 8 days	73	48.7
Delivery mode		
SVD	63	42
C/S	87	58
Position of lines		
Central placed	105	70
Peripheral IV catheter	45	30

*SVD* spontaneous vaginal delivery, *C/S* caesarean section

### Maternal characteristics

The mean age of mothers of the neonates included in the study was (28 ± 6) years with a minimum age of 16 years and maximum 41 years. The number of mothers who resided within the Ho municipality was 52 (34.7%). Most of the mothers were disproportionately Christians, 142 (94.7%) (Table 2).

**Table 2:** Distribution of maternal socio-demographic characteristics

Variable	Count (N) 150	Percent (%)
Age		
≤ 20	20	13.3
21–30	71	47.3
≥ 31	59	39.3
Marital status		
Married	108	72
Single	41	27.3
Divorced	1	0.7
Residence		
Within Ho Municipality	52	34.7
Outside Ho Municipality	98	65.3
Ethnicity		
Ewes	136	90.7
Akan	9	6
Northern descent	5	3.3
Religion		
Christianity	142	94.7
Islam	6	4
Traditional African Belief	2	1.3
Educational level		
No formal education	12	8
Primary	18	12
JSS	59	39.3
SSS	35	23.3
Tertiary	26	17.3
Employment		
Employed	122	81.3
Unemployed	28	18.7

### Common isolates identified

Equal proportions of microorganism were isolated in both early and late onset sepsis. The gram positive isolates identified were *Staphylococcus aureus* and *Staphylococcus epidermidis*. Gram negative organisms include: *Pseudomonas aeruginosa*, *Escherichia coli*, *Enterobacter* species and *Proteus mirabilis* (Table 3). Overall, the commonest microorganism isolated was *Staphylococcus epidermidis* 14 (53.9%), with the greater proportion 9 (69.2%) in early onset sepsis (Table 3). Positive blood cultures were not repeated however, isolates that were considered contaminants by the microbiologist were not included in this study. Lumbar puncture was not done for positive blood cultures in this study.

**Table 3:** Distribution of isolates of blood culture of neonates with sepsis

Isolate	Early Onset Sepsis		Late Onset Sepsis		
	Count	Percentage (%)	Count	Percentage (%)	Total Count (N)
Gram Positive organism					
*Staphylococcus epidermidis*	9	69.2	5	38.5	14
*Staphylococcus aureus*	1	7.7	3	23.1	4
Gram Negative organism					
*Escherichia coli*	1	7.7	0	0	1
*Pseudomonas aeruginosa*	2	15.4	2	15.4	4
*Enterobacter* species	0	0	2	15.4	2
*Proteus mirabilis*	0	0	1	7.7	1
Total	13		13		26

### Antibiotic susceptibility

A total of 44% (66 of 150) of the neonates tested had previously been given antibiotics before their samples were taken.

Based on the antibiotic susceptibility testing of the gram positive organisms isolated from the blood culture, *Staphylococcus epidermidis* showed 100% resistance to ampicillin, and 57% resistance to gentamicin (Table 4).

**Table 4:** Percentage resistance of gram positive organisms isolated from blood culture

Isolate	Count (N)	AMP	GEN	CRX	COT	PEN	FLU
*Staphylococcus epidermidis*	14	100	57	64	100	100	100
*Staphylococcus* *Aureus*	4	100	50	75	50	100	–

*AMP* ampicillin, *GEN* gentamicin, *CRX* cefuroxime, *COT* cotrimoxazole, *PEN* penicillin, *FLU* flucloxacillin

Of the four *Staphylococcus aureus* organisms isolated, there was a 100% (4/4) resistance to ampicillin, 100% (4/4) resistance to penicillin and 50% (2/4) resistance to gentamicin, (Table 4). However, sensitivity to methicillin was not tested in this study.

A total of eight gram negative organisms were identified in this study, namely *Pseudomonas aeruginosa* (4), *Escherichia coli* (1), *Enterobacter* species (2) and *Proteus mirabilis* (1). *Pseudomonas aeruginosa* showed 75% (3/4) resistance to cefuroxime, 50% (2/4) resistance to cefotaxime, and 25% (1/4) resistance to gentamicin (Table 5).

**Table 5:** Percentage resistance of gram negative organisms isolated from blood culture

Isolate	Count (N)	AMP	GEN	CRX	COT	CTR	CTX
Pseudomonas aeruginosa	4	100	25	75	75	25	50
Escherichia Coli	1	100	0	0	100	0	0
Enterobacter species	2	100	50	50	50	50	100
Proteus mirabilis	1	100	100	100	100	0	0

*I* Intermediate susceptibility, *AMP* ampicillin, *GEN* gentamicin, *CRX* cefuroxime, *COT* cotrimoxazole, *CTR* ceftriaxone, *CTX* cefotaxime, *I* intermediate

The percentage resistance of *Escherichia coli* was 100% (1/1) to ampicillin, 100% to cotrimoxazole, 0% to gentamicin, 0% to ceftriaxone and, 0% to cefotaxime (Table 5). *Enterobacter* species showed 100% resistance to ampicillin, 100% resistance to cefotaxime 50% resistance to gentamicin, and 50% resistance to ceftriaxone (Table 5). The resistance of *Proteus mirabilis* was 100% to ampicillin, 100% to cefuroxime, and 0% to cefotaxime (Table 5).

## Discussion

### Common isolates

Culture results in this study shows that 26 (17.3%) of the suspected neonatal sepsis cases were positive. The results indicate that, gram positive organisms (69%, 18/26) has a preponderance over the gram negative organisms (31%, 8/26). This suggests that majority of the infections were transmitted from handling by health care personnel and family members. Since *Staphylococcus epidermidis* and *Staphylococcus aureus* are the major normal flora located on the skin and in the nose respectively, suboptimal hand hygiene by persons who handle neonates, manipulation of peripheral intravenous lines set up on neonates could contribute to the acquisition of these bacteria. Findings from this study, did not correspond to a study done in a Neonatal Intensive Care Unit (NICU) in Bangladesh, where they identified gram negative organisms (78%) to be the most common pathogen of neonatal sepsis [19]. However, in a similar study in Ghana in a tertiary hospital, gram positive organisms had a preponderance over gram negative organisms; similar to findings in this study except that their study had a larger sample size [13].

However, a similar study in a NICU in China, found that gram positive organisms were responsible for a greater proportion of early onset sepsis (83.3%) and late onset sepsis (70%) as compared to gram negative organisms [20], which corroborates the findings of this study. These Findings are also similar to a study in Nigeria which reported a 52.6% proportion of gram positive organisms from blood culture of neonates with sepsis [21].

*Staphylococcus epidermidis* 9 (64%), was the most common isolate identified of all the bacteria in early onset sepsis (EOS). The same findings were reported in a similar study in Ghana, where *Staphylococcus epidermidis* was the most common isolate in both EOS (59.1%) and LOS (52.8%) in a tertiary hospital [13]. Since reports indicate that, organisms causing EOS are mostly transmitted vertically from the colonized genital tract of mothers, or sometimes through the delivery process, the findings suggest that EOS causing organisms could be transmitted by these means.

In a study in Nepal, results revealed that *Staphylococcus epidermidis* accounted for the greatest proportion (57.3%), followed by (28.1%) of *Escherichia coli*, (11.2%) of *Staphylococcus aureus* and (1.1%) of *Pseudomonas aeruginosa* that were isolated in EOS [22]. This is similar to what was observed in this study. Similar reports were given in a study done in Tamale Teaching Hospital in Ghana, where 8 (53.3%) of *Staphylococcus* species, 1 (6.7%) *Escherichia coli* [5] and other gram negative organisms of a total of 15 isolates were identified in EOS.

In the late onset sepsis (LOS) cases, majority of the bacteria identified were *Staphylococcus epidermidis* and *Staphylococcus aureus*. Reports in Nepal, indicate that *Enterobacter* species (15%), *Acinetobacter* species *(12%)* and, *Escherichia coli (12%)* were the commonest isolated gram negative organisms in LOS, which contradicts the findings of this study [23]. In addition, a study done in South Africa, identified *Acinetobacter baumannii*, *Klebsiella pneumoniae* and *Escherichia coli* as the predominant gram negative bacteria, together with few *Pseudomonas aeruginosa* and *Enterobacter* species, which is contrary to findings in this study [24]. Considering that, there is a variation of causative organisms of neonatal sepsis, between geographic regions and facilities, the difference in findings in this study could be acceptable. Bacteriological profile of neonatal sepsis causing organisms may vary among countries [25]. Also, composition of these organisms have changed over the last century because of changing trend of antibiotic use and life style [26].

### Antibiotic susceptibility

Results of antibiotic susceptibility in the present study indicate that, *Staphylococcus epidermidis*, shows 100% resistance to ampicillin, penicillin, flucloxacillin and cotrimoxazole. This is alarming considering that, either ampicillin or penicillin in combination with gentamicin is recommended as first line drugs for empiric treatment of neonatal sepsis. Though *Staphylococcus epidermidis* shows an approximated average (43%) sensitivity to gentamicin, it indicates that its treatment by first line drugs poses a threat to management of neonates. Neonates may spend longer days on antibiotics and stay longer in hospital as well. This confirms reports in an antibiotic susceptibility test carried out on blood cultures from neonates that, Coagulase Negative *Staphylococcus* including *Staphylococcus epidermidis* showed poor sensitivity (13.5%) to ampicillin [27]. In another study, *Staphylococcus epidermidis* showed 100% resistance to ampicillin, penicillin and cotrimoxazole [28], which corresponds to the findings of this study. Thus, results in this study could serve as evidence of increasing resistance to commonly used antibiotics.

*Staphylococcus aureus*, the second gram positive isolate in this study is 100% resistant to ampicillin, and penicillin. The overall high resistance rate exhibited could be attributed to the frequent and unrestricted use of the commonly used antibiotics. Consequently, this could limit future antibiotic choice for treating neonatal infections thereby, affecting survival of septic neonates. In Ghana, resistance rate of 96.4%, and 96.4% to ampicillin, and penicillin respectively was reported [5]; this confirms high resistance rate to these antibiotics identified in this study. Susceptibility to methicillin was not tested in this study, thus sensitivity and resistance to methicillin among neonates was not determined.

We observed a lower resistance (25%) to ceftriaxone and gentamicin. High sensitivity to gentamicin and ceftriaxone by *Pseudomonas aeruginosa* is good for neonatal care. This means that, the use of both drugs in empiric treatment of neonatal sepsis will be effective against *Pseudomonas aeruginosa.* However, the complete resistance to ampicillin, as exhibited by other organisms in this study is devastating. As observed in a study done in India, *Pseudomonas aeruginosa* was 25% resistant to gentamicin, [29] which is comparable to this study.

*Enterobacter* species showed multidrug resistance (100%) to ampicillin, and cefotaxime. Multidrug resistance in the sick newborn is not a desirable experience for the neonate, family or clinical management staff. The reason is that, it leads to increased cost in terms of money and productive time spent at the hospital by family. Additionally, multidrug resistance of infectious organisms could result in unsuccessful treatment leading to death. Though sensitivity to gentamicin and the third generation cephalosporins (cefuroxime and ceftriaxone) are fairly high, it calls for caution and restriction in its use. As observed in Nepal, *Enterobacter* species had a high multidrug resistance rate (100%) to antibiotics including ampicillin and cefotaxime [23], which is consistent with findings in this study.

The only *Escherichia coli* isolated also exhibits a multi drugresistance to ampicillin, and cotrimoxazole. However, there was no susceptibility to gentamicin and the cephalosporin (cefuroxime, ceftriaxone and cefotaxime). *Escherichia coli* also exhibits multidrug resistance like *Pseudomonas aeruginosa* in this study, which poses a threat to neonatal care in this era of increasing antibiotic resistance. These results are partly consistent, and partly disagrees with a similar study done in Indonesia where *Klebsiella pneumoniae*, *Escherichia coli*, *Enterobacter* species and non-fermenting gram negative bacilli were the gram negative organisms isolated; it was reported that, all of them exhibited high resistance to ampicillin, gentamicin and cefotaxime [30].

The single *Proteus mirabilis* organism identified, also exhibits a high level of multidrug resistance but not susceptible to ceftriaxone and cefotaxime. The high level of antibiotic resistance observed is in line with the emerging threat of global antibiotic resistance. One factor accounting for this, could be the indiscriminate use of available antibiotics in the presumptive treatment of neonatal infection. Also, the tendency of clinicians initiating antibiotic therapy before performing blood culture could result in antimicrobial resistance. As observed in this study, blood was obtained from 44% (66 of 150) neonates after the initiation of antibiotic therapy. Thus, it could be a contributing factor to high rates of antibiotic resistance exhibited in this study.

Findings from this study reveal that, all the common isolates of neonatal sepsis show 100% resistance to ampicillin. In addition, all gram positive organisms show 100% resistance to penicillin. However, organisms show appreciable sensitivity to gentamicin, ceftriaxone and cefuroxime. This indicates that “ampicillin/penicillin +gentamicin” combination as the first line of drugs for empiric therapy, needs to be reviewed in terms of ampicillin and penicillin.

### Limitations

This study did not determine the selective pressure factors influencing antibiotic susceptibility, but only determined antibiotic susceptibility. Thus factors associated with high antibiotic resistance in this study are not known. The initiation of antibiotic therapy in some neonates prior to obtaining blood culture sample could have reduced culture positivity, hence affecting prevalence of culture proven sepsis in the study. Methicillin was not contained in the multidisc used for the culture, there sensitivity tests for *S. aureus* could not be carried out.

More infants delivered by C-section were included in the study, therefore, the isolated organism isolated are likely to be different. This is because C-section infections maybe hospital acquired and mainly gram negative.

Also, we did not collect data on UTI among mothers. However, only 3 and 12 case notes of mothers contained information on chorioamnionitis and maternal fever. Unavailability of these variables on majority of mothers was a limitation.

## Conclusion

The prevalence of culture proven neonatal sepsis in this study is 17.3%. Gram positive organisms were the prevalent neonatal sepsis causing organisms in this study. Of the gram positive organisms, *Staphylococcus epidermidis* was the most common isolate, followed by *Staphylococcus aureus*. *Pseudomonas aeruginosa* was the most common isolate among the gram negative organisms, with single isolates each of *Escherichia coli* and *Proteus mirabilis*. Generally, gram negative bacteria exhibit a better susceptibility rate to gentamicin and ceftriaxone, whiles gram positive organisms are more sensitive to gentamicin. Gentamicin as the first line antibiotic for neonatal sepsis is still quite effective. However, the microorganisms isolated in this study demonstrate an increasing resistance rate. All neonatal sepsis causing organisms were completely resistant to ampicillin and penicillin and hence the need to review the empirical treatment for neonatal sepsis.

## Additional file

Additional file 1: Data spreadsheet. (XLSX 25 kb)

## Abbreviations

CoNS: Coagulase negative staphylococcus
EOS: Early onset sepsis
LOS: Late onset sepsis
NICU: Neonatal Intensive Care Unit
NS: Neonatal sepsis
